# Novel mixed ligand coordination compounds of some rare earth metal cations containing acesulfamato/N,N-diethylnicotinamide

**DOI:** 10.3906/kim-2012-47

**Published:** 2021-08-27

**Authors:** Leriman ZEYBEL, Dursun Ali KÖSE

**Affiliations:** 1 Department of Chemistry, Science and Arts Faculty, Hitit University, Çorum Turkey

**Keywords:** Rare-earth metal complexes, acesulfamato, *
N,N-* diethylnicotinamide, structural characterization, thermal analysis, mixed ligand complexes

## Abstract

The mixed ligand coordination compounds containing acesulfamato and *N,N* -diethylnicotinamide biomolecules of some rare earth metal cations (Eu^3+^, Tb^3+^, Ho^3+^, Er^3+^ and Yb^3+^) were synthesized, and their structural properties were investigated. Possible structural formulas have been proposed by determining the chemical composition of molecules (elemental analysis), binding properties (infrared spectroscopy, mass analysis, solid-state UV-vis spectroscopy), thermal degradation properties (TGA / DTA curves). Based on the data collected, it is suggested that rare earth metal cations with a 3+ oxidation state have sextet coordination. The geometries of the structures were thought to be distorted octahedral. The charge balance of the coordination sphere is balanced by a monoanionic acesulfamato located outside the coordination sphere. When the thermal behaviours of the complexes were examined, it was determined that the compounds with Eu^3+^, Tb^3+^, and Yb^3+^ metal cations contained one hydrate water outside the coordination sphere. Hydrate waters do not exist in the Ho^3+^ and Er^3+^ metal cation-centred complexes. At the end of the thermal decomposition analysis of all complex structures, it was determined that they leave the relevant metal oxides in the reaction vessels as final decomposition products.

## 1. Introduction

Studies on the coordination compounds of rare earth metal (REM) cations started later than studies on transition metals. The complex compounds formed by rare earth metal cations with organic ligands have rich chemistry, unique physical properties, different and essential application areas. For this reason, interest in these compounds has been increasing for the last half-century. Due to their characteristic photophysical and magnetic properties, their use in magnetic and optical devices [1–6], sensor systems [7], biological analysis applications [8–10], and medical diagnostic devices [11–14] is increasing.

Rare earth metal cations prefer to form metal complexes with high coordination numbers with hard Lewis-based donors such as F, O, and N due to their strong Lewis acid character and large ionic radii. The carboxylic acids containing strong Lewis donor atoms, such as O and N, and polyaminopolycarboxylic acids are among the most suitable coordination ligands for rare earth metal cations with 3+ oxidation steps and meet the high coordination numbers needed [15,16].

Today, in biological applications, clinical studies, NLO (nonlinear optics) applications, OLED (organic light-emitting diode) applications, MOF (metal-organic framework) compounds [17–19] and even corrosion inhibitors with traditional and toxic chromate-based compounds demonstrated potential for use [20]. On the other hand, the increasing use of rare earth metal compounds in clinical treatments and medicines may raise some negative concerns about their long- and short-term effects in humans and animals. One of the most important physiological effects of rare earth metal cations is their ability to block both voltage-operated and receptor-operated calcium channels [21–23]. Approximately 100 complex structures containing the aminoacid ligament, which is considered an important ligand group for rare earth metal cations, were examined and characterized by X-ray single-crystal analysis [23,24]. Large rare earth metal cations, depending on their radius, generally prefer to form coordination compounds with high coordination numbers (7–10). However, coordination compounds forming six coordinated complex structures with ligands showing strong electron donor properties are rarely reported in the literature [25–29].

**Figure 1:**
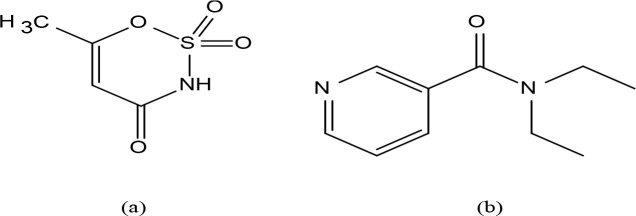
Molecular formula of acesulfamato (a) and N,Ndiethylnicotinamide (b).

Acesulfamato (C_4_H_4_SO_4_NH, HAcs, Figure 1) is an oxathiazinone dioxide discovered by Clauss [30] in 1967 and has been widely used as an artificial sweetener since 1988 [31] after the FDA (Food and Drug Administration) [32].

Acesulfame anion (C_4_H_4_SO_4_N^-^, *acs* ^-^) is an interesting and versatile ion. It is an exciting molecule with donor acidic imino nitrogen, one carbonyl and two sulfonyl oxygen. These donor atoms can exhibit structural diversity by differently coordinating their metal centres. Acesulfame anion, nicotinamide [33,34], N,N-diethylnicotinamide (Fig. 1b) [35], 3-aminopyridine [36], N,N-dimethylethylenediamine [37], 2-aminopyrimidine [38], etc. tend to form crosslinked metal complexes with neutral ligands that have strong electron donating groups. It can also serve as the stabilizing anion of the coordination compounds formed by this type of ligands. In acesulfame metal complexes, via the acidic imino nitrogen atom coordinated to the metal cation [(Co( *acs* )_2_(H_2_O)_4_] [39], via the carbonyl oxygen atom [Ni( *acs* )_2_(H_2_O)_4_] [40] or as in the complex structure of [Cu(ampy)_2_( *acs* )_2_] [35,38], compounds with which a coordination is made through both donor atoms were synthesized.

In this study, coordination compounds with mixed ligands of acesulfame ( *acs* ) and *N,N-* diethylnicotinamide (nikethamide, *dena* ) of the rare earth metal cations Eu^3+^, Tb^3+^, Ho^3+^, Er^3+^, and Yb^3+^ were synthesized. Afterwards, the structural characterizations of the molecules were examined, and their thermal degradation steps were investigated in detail.

## 2. Materials and methods

The chemicals used in synthesis reactions [Eu(ClO_4_)_3_.6H_2_O (europium(III)perchlorate hexahydrate, 50% w/w in aq. soln.), Tb(ClO_4_)_3_ (terbium(III)perchlorate, 50% w/w in aq. soln.), Ho(ClO_4_)_3_ (holmium(III)perchlorate, 50% w/w aq. soln.), Er(ClO_4_)_3_.6H_2_O (erbium(III)perchlorate, 50% w/w in aq. soln.), Yb(ClO_4_)_3_ (ytterbium(III)perchlorate, 50% w/w in aq. soln.), potassium acesulfame and *N,N-* diethylnicotinamide] were obtained from Alfa-Easer reagent grade. 

### 2.1. Synthesis of complexes

**Figure 2:**
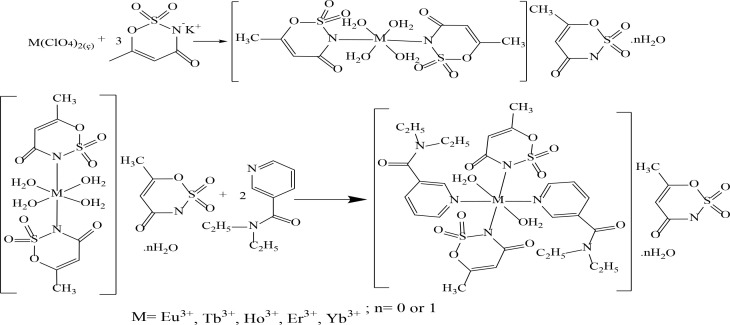
The synthesis reaction of mixed ligand complexes of rare-earth metal-acesulfame/N,N-diethylnicotinamide.

In the synthesis of the complexes, perchlorate salts of 0.01 mol Eu^3+^, Tb^3+^, Ho^3+^, Er^3+^ and Yb^3+^ cations were dissolved in 30 mL of water, 20 mL of the aqueous solution of 0.03 mol of acesulfame-K was added to the solution obtained. The potassium perchlorate (KClO_4_) was expected to settle from the continuously stirred solution. Some cold ethyl alcohol was added to the solution to make the precipitation process better. After precipitation, filtration was carried out, and 50 mL of an aqueous solution of 0.02 mol of nicotinamide was added to the solution. The total solution mixed over a magnetic stirrer for 2 h was stored at room conditions for crystallization. The visualization of two-stage general synthesis reactions of mixed ligand metal-acesulfame/ *N,N-* diethylnicotinamide complexes is given in Figure 2. Elemental analysis results of metal-acesulfame/ *N,N* -diethylnicotinamide complexes are given in Table 1.

**Table 1:** Elemental analysis data of mixed ligand complexes of rare-earth metal-acesulfamato/N,N-diethylnicotinamide.

Complexes	M.W.(g/mol)	Yield(%)	Chemical Analysis (%)Exp. (Theo.)				Colour	Decomp. Temp.(°C)
			C	H	N	S		
[Eu(acs)2(dena)2(H2O)2](acs).H2OC32H46EuN7O17S3	1048.90	57	37.03(36.64)	4.98(4.42)	9.51(9.35)	9.66(9.17)	pale pink	100
[Tb(acs)2(dena)2(H2O)2](acs).H2OC32H46N7O17S3Tb	1055.86	55	36.82(36.40)	5.13(4.39)	9.40(9.29)	9.43(9.11)	pale pink	93
[Ho(acs)2(dena)2(H2O)2](acs)C32H44HoN7O16S3	1043.85	63	36.25(36.82)	5.16(4.25)	9.32(9.39)	8.87(9.22)	pale pink	86
[Er(acs)2(dena)2(H2O)2](acs)C32H44ErN7O16S3	1046.18	60	36.36(36.74)	4.93(4.24)	9.43(9.37)	9.71(9.19)	white	104
Yb(acs)2(dena)2(H2O)2](acs).H2OC32H46N7O17S3Yb	1069.99	50	36.21(35.92)	5.08(4.33)	9.29(9.16)	8.31(8.99)	pale pink	91

### 2.2. Infrared (FT-IR) spectroscopy

**Figure 3:**
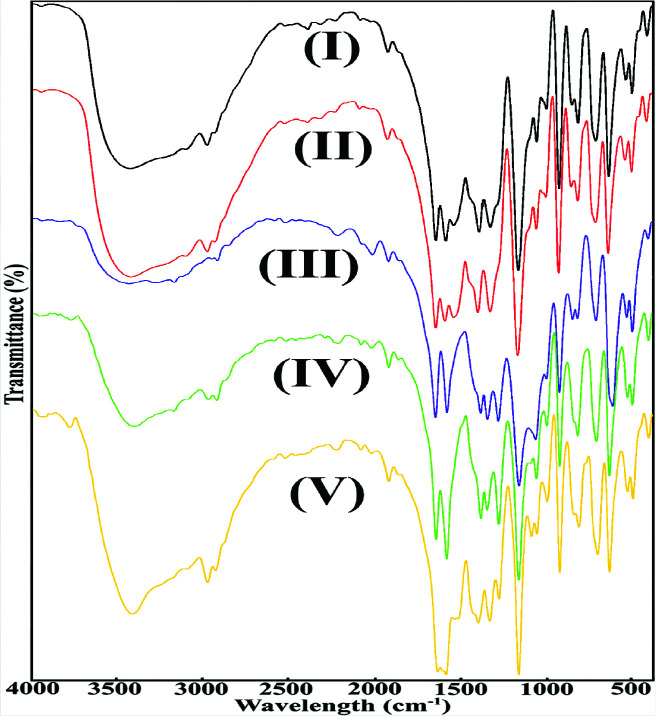
FT-IR spectra of mixed ligand complexes of rare-earth metal-acesulfamato/N,N-diethylnicotinamide.

FT-IR spectra of acesulfamato-diethylnicotinamide complexes of synthesized rare earth metal cations (Eu^3+^, Tb^3+^, Ho^3+^, Er^3+^ and Yb^3^) were recorded in the 4000–400 cm^–^1 range (Figure 3), and characteristic vibration bands were determined, and the relationships between the structures of the complexes and these vibrations were investigated. In order to learn more about the coordination properties of the complexes, the stretching vibrations of the acesulfamate ligand groups such as imine, carbonyl and sulfonyl were compared to the vibrations of the potassium acesulfamato salt, and the changes in the vibrations were examined. The violent and broad peaks seen in the infrared spectra of the complexes in the range of 3650 cm^–1^–2850 cm^–1^ can belong to the aqua –O-H group stress vibrations. Aromatic -C-H stretching vibrations in the structure of the ligands appeared as sharp and severe peaks in the range of 2987 cm^–1^–2979 cm^–1^. There was no shift towards the low region, indicating coordination in stress vibrations attributable to two different carbonyl groups in the structure of the ligands. This situation indicates that (-C=O) groups do not participate in coordination. In the complexes, the peaks, which are thought to belong to the carbonyl group of the acesulfamato ligand, were observed at very close values between 1660 cm^–1^–1650 cm^–1^. The carbonyl ligand of the amide group in the nikethamide ligand appeared in the range of 1615 cm^–1^–1605 cm^–1^. It was determined that the tensile vibrations of the C-N-C groups in the structure of both ligands, which do not coordinate, shift to lower regions than the peaks that can be attributed to these groups. This can be given as evidence that coordination in both ligands takes place over the -N atom in these groups. These coordination connections are also compatible with the literatüre [33–39]. While the stretching vibrations of the acesulfamato ligand belonging to the C-N-C group were observed in the range of 1363 cm^–1^–1356 cm^–1^, the stretching vibrations of the C-N-C group in the pyridine ring of the nicotinamide ligand occurred in the ranges of 1395 cm^–1^–1393 cm^–1^. The difference between the asymmetric and symmetrical stress vibrations of the SO_2_ group can be demonstrated as evidence that the acesulfamato group does not coordinate. The difference between n(SO_2_)_asym_and n(SO_2_)_sym_ strain vibrations remains below 140 cm^–1^ in cases where coordination is not achieved over the SO_2_ group [41–43]. In the complexes of rare earth elements synthesized, this value is in the range of 112 cm^–1^–119 cm^–1^. The peaks that can be attributed to the coordination of the ligands to metal cations are identified. Accordingly, while the coordination of acesulfamato takes place through the anionic -N group, n(M-N)_acs_ stress vibrations occur in the given order of metal cations in the regions of 629 cm^–1^, 653 cm^–1^, 649 cm^–1^, 649 cm^–1^ and 620 cm^–1^, respectively. The vibrations related to the coordination of the n(M-N)_pyrd_ group over the pyridine nitrogen of the nicotinamide ligand were detected in the regions of 675 cm^–1^, 668 cm^–1^, 673 cm^–1^, 673 cm^–1^ and 645 cm^–1^, respectively. The vibration peaks of aqua binding, which can be attributed to the coordination of aqua ligands in the complex structures (M-O), occurred in the regions of 512 cm^–1^, 518 cm^–1^, 516 cm^–1^, 516 cm^–1^ and 516 cm^–1^, respectively. The peaks that can be attributed to the significant binding vibrations of complex structures are summarized in Table 2. 

**Table 2:** Significant FT-IR peaks of mixed ligand complexes of rare-earth metal-acesulfamato/N,N-diethylnicotinamide.

Gruplar	Eu-acs-dena	Tb-acs-dena	Ho-acs-dena	Er-acs-dena	Yb-acs-dena
n(OH)H2O	3560–2850	3560–3380	3650–2850	3650–3150	3550–3150
n(–C–H)	2985	2987	2979	2981	2983
n(C=C)	2222	2221	2227	2228	2229
n(C=O)acs	1660	1650	1655	1654	1652
n(C=O)dena	1615	1613	1610	1605	1607
n(C=C)	1467	1499	1540	1540	1554
n(C–N–C)acs	1357	1356	1357	1357	1363
n(C–N–C)dena	1395	1396	1393	1393	1394
nas(SO2)/ns(SO2)	1289/1177	1286/1172	1314/1199	1314/1195	1319/1203
nas-s	112	114	115	119	116
n(ring)	1098–827	1078–831	1061–834	1060–833	1090–840
ns(CNS)/nas(CNS)acs	1322/939	1331/943	1323/935	1321/934	1309/938
n(C–N)	1007–727	1015–732	1013–735	1014–734	1014–723
n(M–N)acs	629	653	649	649	620
n(M–N)dena	675	668	673	673	645
n(M–O)aqua	512	518	516	516	516

### 2.3. Thermal analysis

Thermal analysis studies of the complex compounds (TG-DTG, DTA) were performed with the Shimadzu DTG-60H system in an inert nitrogen atmosphere (100 mL/min), at a heating rate of 10 °C / min, using a platinum pan using α-Al_2_O_3_ as a reference. The weight reduction caused by volatile products withdrawing from the structures as a result of the decomposition of the compounds was calculated from the TG curves. The reduced weight ratios and metal-ligand ratios from the last remaining degradation products were found.

When the DTG curve of the Eu(III) mixed ligand ( **I** ) complex is examined, it is seen that it degrades in six steps. In the first decomposition step, hydrate water is removed (exp:2.07%; calc:1.72%). In the next dehydration step, two aqua ligands coordinated with the metal are decomposed and removed from the structure (exp:3.34%; calc:3.43%).

The degradation of organic ligands begins by forming 3/2SO_2_ gas of the sulfo group (both coordinated and acting as a counter-ion) from acesulfameto ( *acs* ) molecules (exp:9.02%; calc:9.16%). The fourth degradation step is the step in which organic residues continue to decompose, and the neutral ligand *N,N* -diethylnicotinamide ( *dena* ) begins to decompose together with the acesulfamato ligand (Table 3).

**Table 3:** Thermal analysis data of mixed ligand complexes of rare-earth metal-acesulfamato/N,N-diethylnicotinamide.

Complex		Temperature Range (°C)	DTAmax (°C)	LeavingGroup	Mass Loss(%)		RemainedProduct (%)		Decomp.Product	Colour
					Exp.	Theor.	Exp.	Theor.		
[Eu(C4H4NO4S)2(C10H14N2O)2(H2O)2](C4H4NO4S).H2OC32H46EuN7O17S31048.90 g/mol	1	42–99	89	H2O	2.07	1.72				pale-pink
	2	100–185	144	2H2O	3.34	3.43				
	3	186–220	–214	3/2SO2	9.02	9.16				
	4	269–427	335	2C5H10NO;3C2H3	26.52	26.83				
	5	428–718	450;586	2C5H4N	14.16	14.89				
	6	719–931	737	3C2HNO	15.12	15.74	29.77	28.23	1/2Eu2(SO4)3	black
[Tb(C4H4NO4S)2(C10H14N2O)2(H2O)2](C4H4NO4S).H2OC32H46N7O17S3Tb1055.86 g/mol	1	42–92	80	H2O	1.62	1.71				white
	2	93–170	140	2H2O	3.17	3.41				
	3	171–263	–223; –251	2C5H10NO;3/2SO2	27.67	28.07				
	4	264–451	338	2C5H4N;3C2H3	22.91	22.48				
	5	452–740	477;603	3C2HNO	15.20	15.63	29.43	28.71	1/2Tb2(SO4)3	black
[Ho(C4H4NO4S)2(C10H14N2O)2(H2O)2](C4H4NO4S)C32H44HoN7O16S31043.85 g/mol	1	77–165	87	2H2O	3.42	3.45				pale-pink
	2	166–250	–206	2SO2	12.22	12.27				
	3	251–437	305	2C5H10NO	18.67	19.18				
	4	438–502	–464	SO2; 3CH3; 2C5H4N	25.35	25.41				
	5	503–791	703;772	3C3HO; 3/2N2O	20.88	21.57	17.07	18.10	1/2Ho2O3	black
[Er(C4H4NO4S)2(C10H14N2O)2(H2O)2](C4H4NO4S)C32H44ErN7O16S31046.18 g/mol	1	104–186	146	2H2O	4.04	3.44				pale-pink
	2	185–266	–243	3SO2	19.13	18.37				
	3	267–476	314	2C5H10NO	19.57	19.13				
	4	477–533	–521	2C5H4N	13.86	14.93				
	5	558–877	670;785	3C4H4O; 3/2N2O	24.82	25.83	17.12	18.28	1/2Er2O3	black
Yb(C4H4NO4S)2(C10H14N2O)2(H2O)2](C4H4NO4S).H2OC32H46N7O17S3Yb1069.99 g/mol	1	78–179	146	H2O; 2H2O	5.42	5.05				white
	2	180–285	–243	3SO2; 3C3H4	29.51	29.19				
	3	286–606	404;573	2C5H10NO	19.11	18.72				
	4	607–820	646;764	2C5H4N; 3CO; 3/2N2O	27.97	28.62	17.63	18.42	1/2Yb2O3	black

The next step in the temperature range of 350–450 °C is the step where the last residue of the dena ligand (two C_5_H_4_N) burns away from the structure (exp:14.16%; calc:14.89%).

In the last decomposition phase, it was predicted that the C_2_HNO_2_S in the sphere in the coordination area and the other C_2_HNO_2 _ligand in the counter-ion position were degraded and 1/2 Eu_2_(SO_4_)_3_ remained as the final product (exp:29.77%; calc:28.23%).

When the degradation curve and decay steps of the Tb(III) metal cation-centered complex were examined, it was found that it showed similarities with the Eu(III) metal cation complex. Unlike the Eu(III) complex, it is suggested that in this structure, SO_2_ gas exit does not occur in a separate step, but in the degradation step of the *dena* ligand. It is believed that the sulfate salt [1/2 Tb_2_(SO_4_)_3_; exp:29.43%; calc:28.71%)] remains in the reaction vessel as the final decomposition product in this complex. Details of the degradation steps of the complex are summarized in Table 3. 

The thermal analysis data of the metal cation-centered complex structures of Ho(III) ( **III** ), Er(III) ( **IV** ), and Yb(III) ( **V** ) are quite similar. It is predicted that in the thermal decomposition that occurs in five stages in the Ho(III) and Er(III) complexes, which are thought to not contain hydrate waters, two aqua ligands, which are coordinated in the structures, are firstly removed (exp: 3.42%; calc: 3.45% for Ho(III) and exp: 4.04%; calc: 3.44% for Er(III)). In the second step, it is thought that the *acs* ligand for both complexes begins to decay, giving 2 SO_2_ gas for Ho(III) and 3 SO_2_ gas for Er(III).

It is thought that two groups of C_5_H_10_NO related to the degradation of the *dena* ligand in the next step were removed. In the third decay step of the Ho(III) complex, the remaining SO_2_ group of the *acs* ligand, three CH_3_ residues and the remaining organic residues of the *dena* ligands are removed. In the specified degradation step of the Er(III) complex, organic residues of *dena* ligands move away from the structure (Table 3). It is believed that in the last decay steps of both complexes, the remaining organic residues are burned off and the oxide compounds of the corresponding metal cations remain as the final decomposition product [exp: 17.07%; calc: 18.10% for Ho(III) and exp: 17.12%; calc: 18.28% for Er(III)].

When the DTG curve of the Yb(III) mixed ligand ( **V** ) complex is examined, it is seen that it degrades in four stages. In the first decay phase, it is predicted that a total of three aqua molecules in the structure, one hydrate water and two coordination water, are removed. The next degradation step is the degradation step that can be attributed to the degradation of the sulfonyl groups (3 SO_2_) and organic residues (3 C_3_H_4_) of the *acs* ligands (exp:29.51%; calc: 29.19%). It is suggested that in the third decay step, two C_5_H_10_NO organic residues of *dena* ligands are cleaved (exp: 19.11%; calc: 18.72%). In the last decomposition step, it is claimed that all organic residues in the structure burned away and 1/2 Yb_2_O_3_ oxide compound remained (exp: 17.63%; calc: 18.42%). The compatibility of experimental and calculated weight losses also supports this situation.

**Figure 4:**
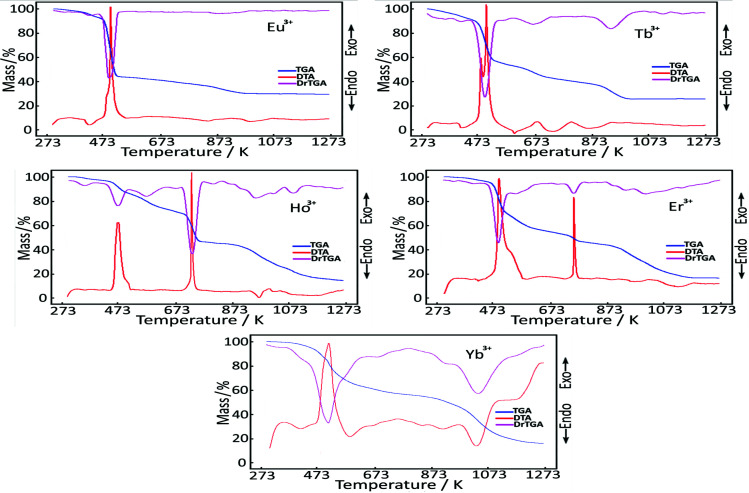
Thermal analysis curves of mixed ligand complexes of rare-earth metal-acesulfamato/N,N-diethylnicotinamide.

The thermal analysis curves of the lanthanide group metal cation complexes are given in Figure 4. Data showing the degradation steps and details are summarized in Table 3. The presence of related metal oxide and sulfate compounds, which are the products of the final degradation step resulting from thermal analysis of metal cation complex compounds, was confirmed by powder x-ray analysis.

### 2.4. Solid-state UV-vis-NIR spectroscopy

**Figure 5:**
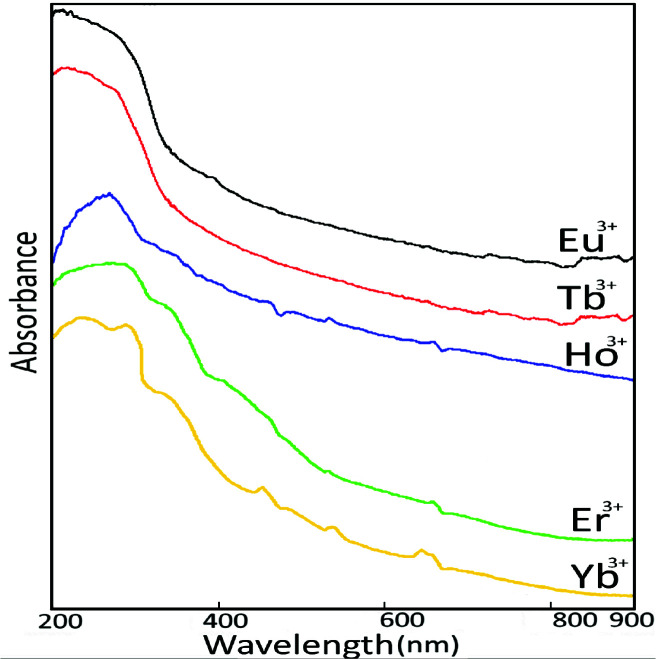
Solid-state UV-vis spectra of rare-earth metalacesulfamato/ N,N-diethylnicotinamide mixed ligand complexes.

When the recorded solid-state UV-VIS-MR spectroscopy curves of lanthanide semi-group metal ions with a charge value of 3+ are examined, a distinct peak in the 850–400 nm range corresponding to the band transition regions of the metals was not observed (Figure 5). The transition of the electrons to high energy levels in the final orbital *“f”* orbitals of the cationic rare earth elements that undergo splitting under UV light was not observed. All complexes are either colourless or almost pale pink in colour. There are breaks that can be interpreted as small vibration peaks in all the synthesized complex structures of only Ho^3+^ metal cation. This situation can be attributed to the increasing electron density in the Ho^3+ ^cation compared to the Eu^3+ ^and Tb^3+ ^cations. The reason why it is not observed in Er^3+^ and Yb^3+ ^metal cation complexes can be explained by the restriction of metal cations due to lanthanide shrinkage. For this reason, the Ho^3+^ metal cation centred complexes have a more off-white or pale pink colour than others.

Since lanthanide ions with 3+ oxidation state will not be oxidized, these transitions may be caused by ligands. Therefore, the high-intensity transition peaks (M-L) occurring in the 400–200 nm region of all metal complexes can be attributed to electron transitions from metal to ligands.

### 2.5. Mass spectroscopy

**Figure 6:**
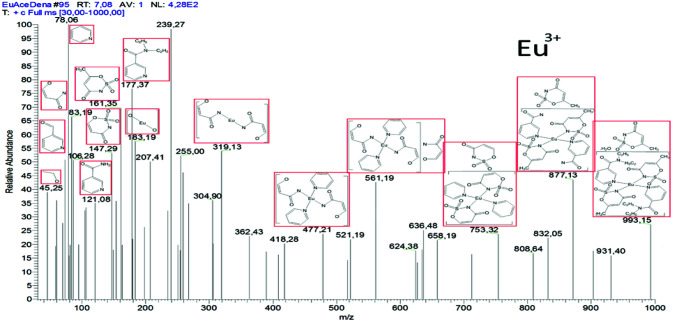
Mass spectra pattern and possible degradation products of mixed ligand complexes of rare-earth metal-acesulfamato/N,N-diethylnicotinamide.

The GC-MS pattern of Eu^3+^ complex and possible cleavage products in the 0–1000 m/z region are given in Figure 6. The mass spectrum patterns of the other four complexes are shown in Figure SF1 in the supplementary file. Similarities were also observed in the recorded GC-MS cleavage products of the coordination compounds thought to have similar structural formulas. Due to the molecular weights of the structures greater than 1000 m/z, the peak values attributable to the molecular ion peaks of the compounds could not be observed. Molecular ion peaks of the dehydrated structure formed due to the removal of the ligand water from the structures were determined. Afterwards, molecular ion peaks were recorded that could be attributed to the residue formed by cleavage of the diethyl groups of the *N,N-* diethylnicotinamide ligand. Molecular ion peaks attributable to acesulfamato, *N,N-* diethylnicotinamide and degradation product nicotinamide molecules in the structure of the complexes were also detected at approximately 161, 177 and 121 m/z regions, respectively. In addition, various peaks that may belong to the leaving products of both acesulfamato and *N,N-* diethylnicotinamide were observed in the mass analysis pattern. Especially peaks belonging to the pyridine ring showed themselves in the 78 m/z area in all complexes. It was also observed in the oxide peaks of the corresponding metal cations, which can be considered as the final cleavage products of the mixed ligand complexes.

## 3. Conclusion 

Complex-ligand complexes of rare earth metal cations (Eu^3+^, Tb^3+^, Ho^3+^, Er^3+^ and Yb^3+^) containing acesulfamato/ *N,N* -diethylnicotinamide were synthesized. Analysis techniques such as elemental analysis, Fourier Transform infrared spectroscopy (FTIR), solid ultraviolet-visible spectroscopy (UV-vis), mass spectroscopy (GC-MS) were studied for the structural properties of the complexes. With the recorded thermal analysis (TGA / DTA) curves, comments were made on the degradation steps of molecules and possible degradation products. According to the chemical composition analysis of the compounds, the metal: ligand1 ( *acs* ): ligand2 ( *dena* ) ratios were claimed to be 1:3:2. 

In the structure of complexes, depending on the steric obstacle, two acesulfamato ligands showed monoanionic-monodentate coordination through acidic -N group, while two *N,N-* diethylnicotinamide ligands provided coordination with the metal through the strong electron donor-N- group in the pyridine ring. The M(III) cations, which are predicted to exhibit sixth coordination, complete the coordination circles with two moles of aqua ligands entering the coordination sphere. Metal cations with characteristic 3+ oxidation steps provide their charge balances with an acesulfamato ligand placed as counter ion outside the coordination sphere. Thermal analysis data confirmed that the Ho(III) and Er(III) complexes did not contain any hydrate water except the coordination sphere. Possible temperature-dependent decomposition products of the complexes were also characterized by thermal analysis method, and the compatibility of experimental and theoretical weight loss was confirmed. Since the molecular weight of the complexes is over 1000 g according to the proposed structural formulas.

The molecular ion peaks were not observed in the mass analysis. Besides, according to the results obtained from elemental analysis data, molecular structure formulas proposed for complex structures are supported.

**Figure 7:**
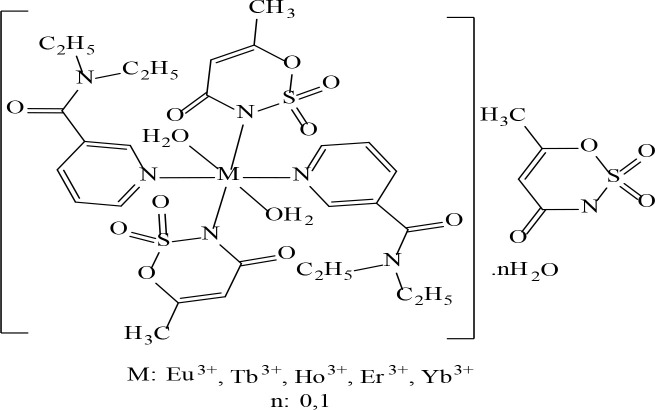
The general structural formula of mixed ligand complexes containing acesulfamato-N,N-diethylnicotinamide ligands.

The comparing results obtained from the analysis and measurements with the similar studies in the current literature, it was deemed appropriate to suggest the molecular structure formula in Figure 7 for complex structures containing acesulfamato- *N,N-* diethylnicotinamide ligands.
